# Supplementary material for: Resolving the full spectrum of human genome variation using Linked-Reads
Source: Genome Res. 2019 Apr;29(4):635–45. doi: 10.1101/gr.234443.118 (PMC6442396; doi:10.1101/gr.234443.118)
Supplement: Supplemental Material [file supp_gr.234443.118_Supplemental_longranger-2.2.zip › longranger-2.2.tar.bz2!/lr2.2-public/lib/stan/cmdstan/stan/doc/api/html/json__handler_8hpp_source.html]

Stan: src/stan/io/json/json\_handler.hpp Source File


|  |  |
| --- | --- |
|  | Stan  2.5.0  probability, sampling & optimization |


- Main Page
- Related Pages
- Namespaces
- Classes
- Files

- File List
- File Members

All Classes Namespaces Files Functions Variables Typedefs Enumerations Enumerator Friends Macros Pages

- src
- stan
- io
- json

json\_handler.hpp

Go to the documentation of this file.

1 #ifndef STAN\_\_IO\_\_JSON\_\_JSON\_HANDLER\_HPP

2 #define STAN\_\_IO\_\_JSON\_\_JSON\_HANDLER\_HPP

3

4 #include <string>

5

6 namespace stan {

7

8  namespace json {

9

15  class json\_handler {

16  public:

17

18  json\_handler() { }

19

20  ~json\_handler() { }

21

25  virtual void start\_text() { }

26

30  virtual void end\_text() { }

31

35  virtual void start\_array() { }

36

40  virtual void end\_array() { }

41

45  virtual void start\_object() { }

46

50  virtual void end\_object() { }

51

55  virtual void null() { }

56

62  virtual void boolean(bool p) { }

63

70  virtual void number\_double(double x) { }

71

77  virtual void number\_long(long n) { }

78

84  virtual void number\_unsigned\_long(unsigned long n) { }

85

91  virtual void string(const std::string& s) { }

92

98  virtual void key(const std::string& s) { }

99  };

100

101  }

102 }

103

104 #endif

stan::json::json\_handler::boolean

virtual void boolean(bool p)

Handle the boolean literal value of the specified polarity.

**Definition:** json\_handler.hpp:62

stan::json::json\_handler::number\_double

virtual void number\_double(double x)

Handle a the specified double-precision floating point value.

**Definition:** json\_handler.hpp:70

stan::json::json\_handler

Abstract base class for JSON handlers.

**Definition:** json\_handler.hpp:15

stan::json::json\_handler::end\_array

virtual void end\_array()

Handle the end of an array.

**Definition:** json\_handler.hpp:40

stan::json::json\_handler::null

virtual void null()

Handle the null literal value.

**Definition:** json\_handler.hpp:55

stan::json::json\_handler::number\_unsigned\_long

virtual void number\_unsigned\_long(unsigned long n)

Handle a the specified unsigned long integer value.

**Definition:** json\_handler.hpp:84

stan::json::json\_handler::key

virtual void key(const std::string &s)

Handle the specified object key.

**Definition:** json\_handler.hpp:98

stan::json::json\_handler::end\_text

virtual void end\_text()

Handle the the end of the text.

**Definition:** json\_handler.hpp:30

stan::json::json\_handler::string

virtual void string(const std::string &s)

Handle the specified string value.

**Definition:** json\_handler.hpp:91

stan::json::json\_handler::start\_object

virtual void start\_object()

Handle the start of an object.

**Definition:** json\_handler.hpp:45

stan::json::json\_handler::start\_array

virtual void start\_array()

Handle the start of an array.

**Definition:** json\_handler.hpp:35

stan::json::json\_handler::~json\_handler

~json\_handler()

**Definition:** json\_handler.hpp:20

stan::json::json\_handler::start\_text

virtual void start\_text()

Handle the the start of the text.

**Definition:** json\_handler.hpp:25

stan::json::json\_handler::end\_object

virtual void end\_object()

Handle the end of an object.

**Definition:** json\_handler.hpp:50

stan::json::json\_handler::json\_handler

json\_handler()

**Definition:** json\_handler.hpp:18

stan::json::json\_handler::number\_long

virtual void number\_long(long n)

Handle a the specified long integer value.

**Definition:** json\_handler.hpp:77


---

[ Stan Home Page ]

*© 2011–2014,
Stan Development Team.*
